# Pulmonary Tc^99m^-PSMA uptake in asymptomatic COVID-19 patient: incidental finding in SPECT/CT study

**DOI:** 10.1186/s43055-022-00750-y

**Published:** 2022-03-15

**Authors:** Forough Kalantari, Reza Vali, Elham Kalantari, Ghasemali Divband

**Affiliations:** 1 Department of Nuclear Medicine, School of Medicine Hazrat-e-Rasool General Hospital, Iran University of Medical Science, Tehran, Iran; 2 The Hospital for Sick Children, University of Toronto, Toronto, ON Canada; 3 Department of Pulmonology, School of Medicine, Isfahan University of Medical Science, Isfahan, Iran; 4 Department of Nuclear Medicine, Jam Hospital, Tehran, Iran

**Keywords:** ^99m^Tc-PSMA, COVID-19, Prostate cancer

## Abstract

**Background:**

As there are comparative studies between ^68^Ga-PSMA and ^99m^Tc-PSMA and spectrum of PSMA expression, this is the first case report that notifies distribution of ^99m^Tc-PSMA on COVID-19 pneumonia era on the literature.

Case presentation

An asymptomatic 70-Y-old male who is known case of prostate adenocarcinoma underwent initial staging. SPECT/CT of the chest region reveals bilateral peripheral multifocal ground glass opacities which shows ^99m^Tc-PSMA uptake. Diagnosis of corona virus was confirmed by positive RT-PCR.

**Discussion:**

Unexclusive role of radiotracers in nuclear medicine has an importance for wide range of applications. Comparison between ^68^Ga-PSMA and ^99m^Tc-PSMA in detection of metastatic disease in prostate cancer is also under evaluation.

**Conclusions:**

This case implicates possible role of PSMA imaging in inflammation/infection process as well as necessity for lung review in hybrid imaging especially during this recent pandemic.

## Background

A spectrum of PSMA expression in benign and malignant findings such as infectious/inflammatory process was reported [1]. There are few reports for ^68^Ga-PSMA uptake in COVID-19 pneumonia [2]. But there is no previously reported case of ^99m^TC-PSMA on COVID-19 as our knowledge. It is also decisive for nuclear medicine physician to know the other possible reasons of ^99m^TC-PSMA uptake for the best interpretation.

## Case presentation

A 70-Y-old male who have biopsy proven prostate adenocarcinoma with Gleason score of 6 (3 + 3) and PSA level 36 ng/ml underwent a ^99m^Tc-PSMA SPECT-CT for initial staging.

A dose of 20 mci (740 MBq) was injected intravenously, and 4 h later, whole-body images and thoracoabdominopelvic SPECT/CT images were performed using the Siemens Symbia Intevo SPECT/CT dual-head variable angle gamma camera.

**Fig. 1:**
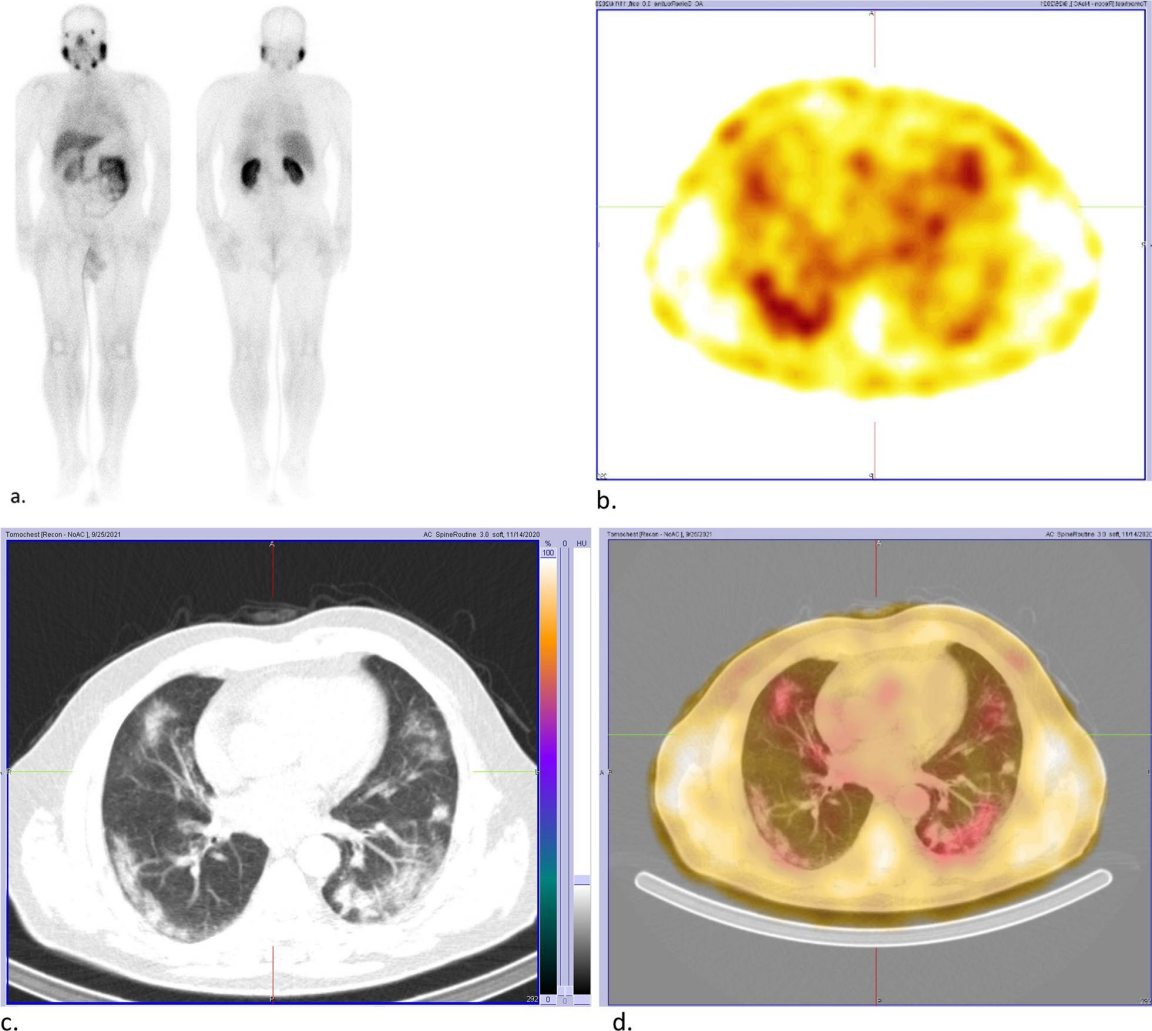
**a** Whole-body 99mTc-PSMA study with faint uptake in the chest region as well as physiologic distribution of radiotracer. **b** SPECT reveals mild peripheral 99mTC-PSMA uptake in the lung fields. **c** CT shows multifocal, bilateral and peripheral ground-glass opacities in lungs. **d** Fused SPECT/CT shows the PSMA uptake is matched by ground-glass opacities in the lung (left lung COVID-related consolidation also shows the same uptake just it is not apparent in this cut due to respiratory motion misregistration)

SPECT/CT scan images revealed heterogeneous PSMA uptake in prostate lobes consistent with previously known malignancy. An incidental finding of multifocal, bilateral and peripheral ground-glass opacities in lungs with mild ^99m^TC-PSMA uptake are noticed (Fig. 1). Otherwise, no abnormal PSMA avid lesions are seen throughout the body.

Regarding viral outbreak, the first diagnosis is COVID-19 pneumonia. Although the patient had no any respiratory symptoms, RT-PCR was performed and the result was positive.

## Discussion

Unexclusive role of radiotracers in nuclear medicine has an importance in wide range of applications. Not to misinterpret, every 99mTc-PSMA uptake in lung fields as metastasis in staging of a prostate adenocarcinoma is crucial. Comparison between 68Ga-PSMA and 99m Tc-PSMA in detection of metastatic disease in prostate cancer is still under evaluation [3, 4]. There are few case reports of incidental detection of COVID-19-associated pneumonia by different tracers on nuclear medicine examinations such as 68Ga-PSMA PET/CT, 18F-FDG PET/CT and thyroid scintigraphy [5–8].

## Conclusion

This case implicates the possible role of PSMA imaging in active inflammatory/infectious process. This may be useful for evaluation of response to treatment of inflammatory disease. Also, as many COVID-19 patients are asymptomatic, it is necessary for nuclear medicine departments to review lung window filed in hybrid imaging even in unrelated pathologies.

## Data Availability

The datasets used and/or analyzed during the current study are available from the corresponding author on reasonable request.

## Abbreviations

68Ga-PSMA: 68 Gallium-prostate-specific membrane antigen
99mTc-PSMA: Technetium-99m-prostate-specific membrane antigen
COVID-19: Coronavirus disease
SPECT/CT: Single-photon emission computed tomography/computed tomography
PSA: Prostate-specific antigen
MBq: Megabecquerels
RT-PCR: Reverse transcription polymerase chain reaction
18F-FDG: 18Florine–fluorodeoxyglucose
PET/CT: Positron emission tomography/computed tomography

## References

[1] De Galiza BF, Queiroz MA, Nunes RF, Costa LB, Zaniboni EC, Marin JFG, Cerri GG, Buchpiguel CA (2020). Nonprostatic diseases on PSMA PET imaging: a spectrum of benign and malignant findings. Cancer Imaging.

[2] Morón S, González E, Rojas J (2020). 68Ga-PSMA PET/CT with incidental finding of COVID-19 in an asymptomatic patient. Clin Nucl Med.

[3] Fallahi B, Khademi N, Karamzade-Ziarati N, Fard-Esfahani A, Emami-Ardekani A, Farzanefar S, Eftekhari M, Beiki D (2021). 99mTc-PSMA SPECT/CT versus 68Ga-PSMA PET/CT in the evaluation of metastatic prostate cancer. Clin Nucl Med.

[4] Albalooshi B (2020). Direct comparison of 99mTc-PSMA SPECT/CT and 68Ga-PSMA PET/CT in patients with prostate cancer. Asia Ocean J Nucl Med Biol.

[5] Stasiak CES, Cardoso FR, de Almeida SA, Rosado-de-Castro PH (2021). Incidental finding of COVID-19 infection after [68Ga]Ga-PSMA-11 PET/CT imaging in a patient with prostate cancer. Eur J Nucl Med Mol Imaging.

[6] Ali SA, Abdelkawi MM (2020). Incidentally recognized COVID-19 pneumonia in routine oncologic 18F-FDG PET/CT examinations: a local experience during pandemic era. Egypt J Radiol Nucl Med.

[7] Jafari F, Alavi M (2021). Incidental detection of COVID-19 associated pneumonia by [99mTc]UBI scintigraphy. Nucl Med Rev Cent East Eur.

[8] Tulchinsky M, Fotos JS, Slonimsky E (2020). Incidental CT findings suspicious for COVID-19-associated pneumonia on nuclear medicine examinations: recognition and management plan. Clin Nucl Med.

